# Predicting gingival embrasure risk after invisible orthodontics using multimodal data and machine learning

**DOI:** 10.2340/aos.v85.46562

**Published:** 2026-07-22

**Authors:** Haiyan Wang, Hanfei Shi, Liping Fan, Zhang Sun, Xuelian Xiu, Hui Yuan

**Affiliations:** a Department of Dentistry, Hongqi Hospital Affiliated to Mudanjiang Medical University, Mudanjiang, China; b Department of Medical Insurance, Hongqi Hospital Affiliated to Mudanjiang Medical University, Mudanjiang, China; c School of Stomatology and School of Basic Medical Sciences, Mudanjiang Medical University, Mudanjiang, China

**Keywords:** Clear aligner therapy, gingival embrasure, risk prediction, machine learning, multimodal data

## Abstract

**Objective:**

To develop and validate a risk prediction model for gingival embrasures after clear aligner therapy using multimodal oral data.

**Methods:**

A retrospective study of 340 patients (December 2022–June 2025) was randomly divided into training (*n* = 238) and validation (*n* = 102) sets (7:3). Univariate analysis, multivariate logistic regression, and least absolute shrinkage and selection operator regression were applied to identify independent risk factors. Three machine learning models – random forest (RF), logistic regression, and support vector machine – were constructed based on seven core variables. Model performance was assessed using area under the receiver operating characteristic curve (AUC), calibration curves, decision curve analysis, and Shapley Additive Explanations (SHAP) values for interpretability.

**Results:**

No significant baseline differences existed between sets (*p* > 0.05). Seven indicators were identified (*p* < 0.05). Multivariate analysis confirmed percentage of bleeding on probing-positive sites, interproximal alveolar bone height, and relative movement of adjacent teeth at target site as independent risk factors, while gingival thickness, proximal contact area, interdental papilla height, and buccal/lingual bone plate thickness were protective factors (*p* < 0.05). The RF model performed best: training AUC = 0.849 (95% CI: 0.786–0.912), validation AUC = 0.815 (95% CI: 0.720–0.910), with good calibration and net benefit. SHAP analysis highlighted gingival thickness and interproximal alveolar bone height as key predictors.

**Conclusion:**

A risk prediction model for post-clear aligner gingival embrasures was successfully developed and validated using multimodal oral data, with RF as the optimal algorithm. The model exhibits good discrimination, calibration, and clinical utility, which can be used as an objective auxiliary tool for individualized risk prediction following clear aligner therapy and supplement traditional clinical empirical judgment.

## Introduction

Adverse changes in the gingival embrasure following clear aligner treatment, such as black triangles and gingival papilla recession, have become critical clinical issues affecting orthodontic esthetic outcomes and patient satisfaction. Studies have reported that the incidence of gingival embrasure risk after clear aligner therapy ranges from 20 to 40%, which not only compromises periodontal health but also leads to food impaction, speech disorders, and esthetic defects, thereby imposing psychological burdens on patients [1, 2]. Although conventional risk assessment methods based on traditional factors such as periodontal status and tooth morphology exist in clinical practice, their predictive accuracy remains significantly limited. Specifically, multidimensional factors – including anatomical differences in periodontal soft and hard tissues, orthodontic treatment design parameters, and patient oral hygiene behaviors – have not been systematically integrated [3, 4]. For example, gingival thickness and alveolar bone plate thickness jointly determine the supportive foundation of the gingival papilla, and patients with thin gingiva and thin bone plates are more prone to papillary recession [5]. Furthermore, the contact area of the proximal surface and the interproximal papilla height directly influence the initial filling status of the gingival embrasure. Moreover, excessive relative movement of adjacent teeth at the target site during aligner treatment may cause the gingival papilla attachment to lag behind tooth displacement, resulting in interproximal gaps [6]. However, how these anatomical features, orthodontic mechanical parameters, and periodontal inflammatory status interact to jointly influence adverse embrasure changes remains unclear. Traditional univariate analysis or multivariate regression models are insufficient to capture and integrate such cross-dimensional, nonlinear complex relationships. In recent years, the widespread adoption of intraoral scanning technology and cone-beam computed tomography has enabled the integration of multimodal data for accurate prediction. Machine learning algorithms, with their powerful pattern recognition and high-dimensional feature processing capabilities, have opened new avenues for constructing risk prediction models that transcend traditional methods by integrating multidimensional factors encompassing anatomy, biomechanics, and inflammation. These algorithms have demonstrated superior performance in orthodontics, periodontics, and related fields [7]. Given this background, the present study defines ‘adverse gingival embrasure changes after clear aligner treatment’ as the clear clinical endpoint. The primary objective is to systematically collect multidimensional data – including periodontal soft tissue parameters, alveolar bone anatomical indices, orthodontic treatment mechanical parameters, and oral hygiene status – through a retrospective study. Consequently, this study aims to construct and validate a risk prediction model that integrates multimodal data and multidimensional factors using machine learning algorithms. The ultimate goal is to achieve individualized and precise risk stratification for gingival embrasure changes in clear aligner patients, thereby providing a scientific decision-making basis and practical tool for early identification of high-risk individuals, optimization of treatment design, and formulation of targeted preventive measures.

## Materials and methods

### Study subjects

This study was designed as a single-center, retrospective, observational study. A total of 340 patients who completed clear aligner treatment in the Department of Orthodontics at our hospital between December 2022 and June 2025 were retrospectively enrolled. Sample size calculation was performed using Power Analysis and Sample Size (PASS) 2021 software based on the expected incidence rate of adverse gingival embrasure changes after clear aligner treatment, which was set at 25–35% according to previous studies [2, 8], combined with the feature number requirements of the proposed multimodal data prediction model (involving least absolute shrinkage and selection operator [LASSO] regression, random forest [RF], and support vector machine). With a two-tailed significance level α = 0.05, power (1-β) = 80%, and an anticipated 10% rate of incomplete data, the minimum required sample size was determined to be 260 participants. The actual enrolled sample size in this study (340 participants) exceeded the minimum requirement. The EPV (events per variable) was calculated as 10.29 (72 positive events/7 core variables), which met the standard of EPV ≥ 10 for machine learning models.

#### Inclusion criteria

(1) age ≥ 18 years; (2) completion of a full course of clear aligner treatment (≥ 10 aligner steps); (3) completion of both pre- and post-treatment intraoral scanning and cone-beam computed tomography with complete imaging data.

#### Exclusion criteria

(1) pre-existing obvious black triangles or severe periodontitis (periodontal probing depth ≥ 6 mm, clinical attachment loss ≥ 3 mm) before treatment; (2) presence of severe systemic diseases (e.g. uncontrolled diabetes mellitus, osteoporosis, and coagulation disorders) that may affect periodontal tissue remodeling; (3) pregnant or lactating women; (4) > 20% missing data for key outcome indicators in medical records; and (5) interruption of treatment or loss to follow-up during the treatment course.

### Data collection

Multidimensional predictor variables were systematically collected by integrating data from the hospital electronic medical record system, oral digital scanning database, cone-beam computed tomography image library, and structured clinical examination records. The collected data included:

1. Basic information and systemic factors: age, sex, smoking history (pack-years), and history of systemic diseases.
2. Oral hygiene and periodontal status: percentage of bleeding on probing (BOP)-positive sites (%), simplified calculus index, and mean gingival index derived from clinical examination records.
3. Tooth morphology and alignment characteristics: pretreatment intraoral scan models (Standard Tessellation Language format) were imported into digital analysis software (e.g. 3Shape and Geomagic) to measure proximal contact area (mm²), axial crown convexity (degrees), tooth rotation angle (degrees), interproximal papilla height (mm), baseline gingival embrasure area (mm²), and proximal contact opening angle (degrees).
4. Periodontal bone tissue and anatomical structure: pretreatment cone-beam computed tomography images were used to measure interproximal alveolar bone height (distance from the cementoenamel junction, mm), interproximal alveolar bone density (Hounsfield units), interradicular bone height (mm), buccal/lingual bone plate thickness (mm), and distance from the proximal contact point to the alveolar crest (mm).
5. Treatment-related indices: total number of aligner steps, planned relative movement of adjacent teeth at the target position (mm), planned intrusion/extrusion amount (mm), and aligner gingival margin configuration (low-coverage vs. high-coverage type) extracted from treatment planning software (e.g. ClinCheck).

All data were independently extracted and entered into a dedicated electronic data capture system by two trained research assistants, followed by consistency verification. The intra-observer and inter-observer intraclass correlation coefficient (ICC) of all measurement indicators were calculated, with all ICC > 0.85, indicating good measurement repeatability.

### Outcome definition

Referring to domestic and international clinical diagnostic criteria for black triangles and gingival papilla recession [9, 10] and considering the retrospective nature of the data in this study, all outcome evaluations were performed immediately after aligner removal upon completion of the full treatment course. The primary outcome was explicitly defined as ‘adverse gingival embrasure change after clear aligner treatment’. The event group comprised patients who developed adverse gingival embrasure changes confirmed by both clinical and digital model assessments after treatment, meeting at least one of the following criteria: (1) development of black triangles after treatment, defined as incomplete filling of the interproximal space by the gingival papilla, a ratio of the distance from the contact point to the alveolar crest to the distance from the contact point to the papilla tip > 0.5, and black triangle height ≥ 1.0 mm; (2) gingival papilla recession ≥ 0.5 mm; or (3) increase in gingival embrasure area ≥ 30% compared with baseline. The non-event group consisted of patients who, during the same follow-up period, did not exhibit any adverse gingival embrasure changes meeting the above definitions or whose changes did not reach the diagnostic threshold. Thus, the event group represented patients with clinically significant adverse gingival embrasure changes, while the non-event group represented those without such changes. All potential endpoint events were adjudicated following a standardized procedure: initial automated screening based on measurement indices, followed by independent review and adjudication by two research physicians blinded to baseline patient characteristics using complete digital models and clinical records. In case of disagreement, a third senior orthodontist or periodontist served as the arbitrator.

### Statistical analysis

Statistical analyses were performed using Statistical Package for the Social Sciences 26.0, R 4.3.0, and Python 3.9 software. Continuous variables with normal distribution were presented as mean ± standard deviation (x ± s), and intergroup comparisons were conducted using independent samples *t*-tests. Categorical variables were presented as counts (percentages) (*n* [%]), and intergroup comparisons were performed using the χ² test or Fisher’s exact test. The total sample was randomly divided into a training set and a validation set at a ratio of 7:3. Meanwhile, 10-fold cross-validation was used for LASSO regression and model parameter tuning, and 1000-times Bootstrap resampling was performed for calibration curve analysis to improve result stability. In the training set, univariate analysis was first performed to screen for variables associated with the outcome (*p* < 0.05). Subsequently, LASSO regression was used for variable compression and feature selection. Based on the selected variables, a multivariate binary logistic regression model and RF and support vector machine models were constructed. The discriminative ability of the models was evaluated using the area under the receiver operating characteristic curve (AUC). Calibration was assessed using calibration curves (bootstrap method with 1000 resampling iterations) and the Brier score. Clinical net benefit was evaluated using decision curve analysis (DCA). The optimal prediction model was determined by comparing model performance in the validation set. Additionally, a nomogram was constructed based on the optimal machine learning model (RF) to visualize the risk prediction model, allowing graphical calculation of individual risk probabilities for gingival embrasure changes. The Delong test was employed to compare differences in AUC values between different models, with *p* < 0.05 considered statistically significant. Calibration of the nomogram was assessed using calibration curves with bootstrap resampling (1000 iterations). Finally, Shapley additive explanation (SHAP) values were used to evaluate the global and local interpretability of the optimal machine learning model, thereby elucidating the direction and magnitude of each feature’s contribution. All tests were two tailed, and a *p*-value < 0.05 was considered statistically significant.

## Results

### Comparison of baseline characteristics between the training set and validation set

In the training set, 72 patients (30.3%) were classified as the event group (patients who developed adverse gingival embrasure changes) and 166 patients (69.7%) as the non-event group. In the validation set, 31 patients (30.4%) were in the event group and 71 patients (69.6%) in the non-event group. No statistically significant differences in baseline characteristics were observed between the training set and the validation set (*p* > 0.05), indicating balanced dataset partitioning and good comparability between the two sets (Table 1).

**Table 1:** Comparison of baseline characteristics between the training set and the validation set.

Variable	Training set (*n* = 238)	Validation set (*n* = 102)	*t*/*χ*²	*p*
Age (years)	32.45 ± 10.23	32.67 ± 10.05	0.183	0.855
Gingival thickness (mm)	1.08 ± 0.31	1.06 ± 0.29	0.556	0.579
Percentage of BOP-positive sites (%)	18.75 ± 12.34	19.02 ± 12.67	0.183	0.855
Simplified calculus index	0.86 ± 0.52	0.84 ± 0.50	0.329	0.743
Mean gingival index	0.74 ± 0.48	0.76 ± 0.47	0.354	0.723
Proximal contact area (mm²)	6.23 ± 1.56	6.18 ± 1.52	0.273	0.785
Axial crown convexity (degrees)	28.45 ± 4.32	28.67 ± 4.28	0.432	0.666
Distance from contact point to alveolar crest (mm)	3.78 ± 0.95	3.81 ± 0.92	0.269	0.788
Tooth rotation angle (degrees)	12.34 ± 8.56	12.15 ± 8.23	0.190	0.850
Interdental papilla height (mm)	3.45 ± 0.87	3.42 ± 0.85	0.293	0.769
Interproximal alveolar bone height	1.56 ± 0.64	1.58 ± 0.62	0.267	0.790
Proximal alveolar bone density (HU)	245.67 ± 78.34	248.12 ± 76.89	0.266	0.791
Inter-radicular bone height (mm)	4.23 ± 1.12	4.19 ± 1.08	0.305	0.761
Buccal/lingual bone plate thickness (mm)	0.67 ± 0.23	0.68 ± 0.22	0.372	0.710
Total number of aligner steps (steps)	28.45 ± 12.34	27.89 ± 12.05	0.386	0.670
Relative movement of adjacent teeth at target site (mm)	2.34 ± 1.12	2.38 ± 1.09	0.304	0.761
Aligner gingival margin design			0.001	0.981
Low-coverage type	142 (59.66)	61 (59.80)		
High-coverage type	96 (40.34)	41 (40.20)		
Planned intrusion/extrusion amount (mm)	0.85 ± 0.67	0.82 ± 0.65	0.382	0.703
Baseline gingival embrasure area (mm²)	4.56 ± 1.34	4.52 ± 1.30	0.255	0.799
Contact point opening angle (degrees)	15.67 ± 4.23	15.45 ± 4.18	0.441	0.660

BOP: bleeding on probing.

### Univariate analysis of factors associated with gingival embrasure changes after clear aligner treatment

The univariate analysis revealed statistically significant differences between the two groups in the following seven core indicators (*p* < 0.05): gingival thickness, percentage of BOP-positive sites, proximal contact area, interdental papilla height, interproximal alveolar bone height, buccal/lingual bone plate thickness, and relative movement of adjacent teeth at the target site. Patients in the event group exhibited thinner gingiva, a higher percentage of BOP-positive sites, smaller proximal contact area, shorter interdental papilla height, greater interproximal alveolar bone height (indicating more bone loss), thinner buccal/lingual bone plates, and greater relative movement of adjacent teeth at the target site, with all differences reaching statistical significance (*p* < 0.05). In contrast, no statistically significant differences were observed between the two groups for the remaining indicators (*p* > 0.05) (Table 2).

**Table 2:** Univariate analysis of factors associated with gingival embrasure changes after clear aligner treatment.

Variable	Event group (*n* = 72)	Non-event group (*n* = 166)	*t*/*χ*²	*p*
Age (years)	32.67 ± 10.34	32.15 ± 10.18	0.360	0.719
Gingival thickness (mm)	0.89 ± 0.22	1.16 ± 0.29	7.064	0.001
Percentage of BOP-positive sites (%)	24.56 ± 13.21	16.23 ± 11.45	4.917	0.001
Simplified calculus index	0.87 ± 0.53	0.84 ± 0.51	0.412	0.681
Mean gingival index	0.76 ± 0.49	0.73 ± 0.48	0.440	0.660
Proximal contact area (mm²)	5.52 ± 1.23	6.71 ± 1.38	6.309	0.001
Axial crown convexity (degrees)	28.34 ± 4.35	28.49 ± 4.30	0.246	0.806
Distance from contact point to alveolar crest (mm)	3.80 ± 0.96	3.71 ± 0.94	0.674	0.501
Tooth rotation angle (degrees)	12.87 ± 8.67	12.24 ± 8.52	0.521	0.603
Interdental papilla height (mm)	2.99 ± 0.76	3.69 ± 0.79	6.351	0.001
Interproximal alveolar bone height	2.02 ± 0.58	1.40 ± 0.52	6.971	0.001
Proximal alveolar bone density (HU)	235.12 ± 78.56	250.89 ± 78.23	1.427	0.155
Inter-radicular bone height (mm)	4.12 ± 1.13	4.34 ± 1.11	1.397	0.164
Buccal/lingual bone plate thickness (mm)	0.52 ± 0.18	0.73 ± 0.21	7.388	0.001
Total number of aligner steps (steps)	30.14 ± 12.56	27.89 ± 12.31	1.287	0.199
Relative movement of adjacent teeth at target site (mm)	2.89 ± 1.21	2.10 ± 0.98	5.309	0.001
Aligner gingival margin design			0.008	0.928
Low-coverage type	43 (59.72)	99 (59.64)		
High-coverage type	29 (40.28)	67 (40.36)		
Planned intrusion/extrusion amount (mm)	0.92 ± 0.68	0.83 ± 0.66	0.958	0.339
Baseline gingival embrasure area (mm²)	4.78 ± 1.36	4.48 ± 1.33	1.588	0.114
Contact point opening angle (degrees)	16.12 ± 4.25	15.45 ± 4.22	1.123	0.263

BOP: bleeding on probing.; HU: Hounsfield Unit

### Multivariate logistic regression analysis of factors associated with gingival embrasure changes after clear aligner treatment

**Figure 1:**
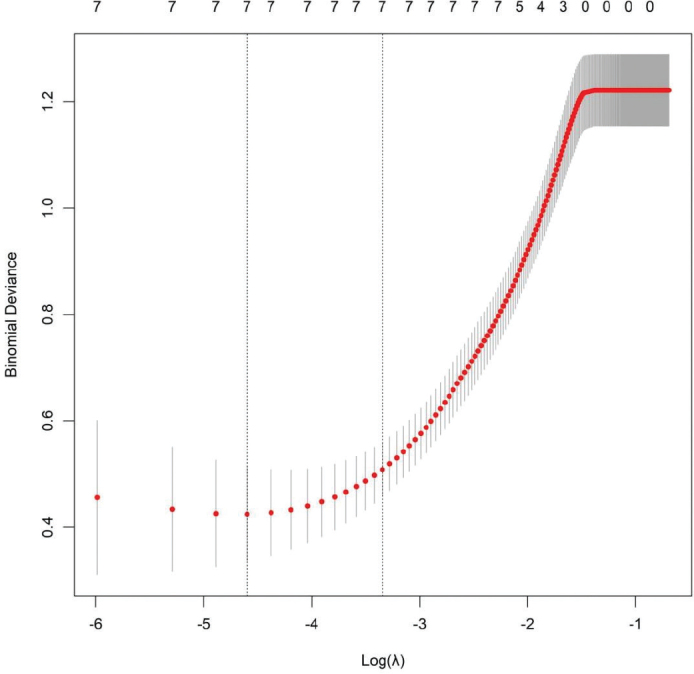
Least absolute shrinkage and selection operator (LASSO) regression plot.

With gingival embrasure risk after clear aligner treatment as the dependent variable (event group = 1, non-event group = 0), all seven indicators that were statistically significant in the univariate analysis (gingival thickness, percentage of BOP-positive sites, proximal contact area, interdental papilla height, interproximal alveolar bone height, buccal/lingual bone plate thickness, and relative movement of adjacent teeth at the target site) were included in the LASSO regression for variable selection (variable assignments are shown in Supplemental Table 1). The optimal variables were selected using 10-fold cross-validation with the λ-1se criterion. As shown in Figure 1, according to the λ-1se criterion of 10-fold cross-validation, the regression coefficients of all seven predictive variables were not compressed to zero, so all variables were retained and incorporated into the multivariate logistic regression model. The results of the multivariate logistic regression analysis (Table 3) indicated that increased gingival thickness, increased proximal contact area, increased interdental papilla height, and increased buccal/lingual bone plate thickness were protective factors against gingival embrasure risk (Odds Ratio (OR) < 1). Conversely, a higher percentage of BOP-positive sites, greater interproximal alveolar bone height (indicating more bone loss), and greater relative movement of adjacent teeth at the target site were risk factors for gingival embrasure risk (OR > 1).

**Table 3:** Multivariate logistic regression analysis of factors associated with gingival embrasure changes after clear aligner treatment.

Indicator	*β*	SE	Wald	*p*	OR	95% CI
Gingival thickness	-4.653	1.130	16.961	0.001	0.010	0.001~0.087
Percentage of BOP-positive sites	0.087	0.026	11.602	0.001	1.091	1.038~1.147
Proximal contact area	-0.784	0.202	15.012	0.001	0.457	0.307~0.679
Interdental papilla height	-1.324	0.414	10.222	0.001	0.266	0.118~0.599
Interproximal alveolar bone height	1.559	0.575	10.833	0.001	4.191	1.926~6.868
Buccal/lingual bone plate thickness	-4.630	1.367	11.479	0.001	0.010	0.001~0.142
Relative movement of adjacent teeth at target site	0.553	0.256	4.677	0.031	1.739	1.053~2.870

BOP: bleeding on probing.

### Evaluation of machine learning model performance

Grid search combined with 10-fold cross-validation was used for hyperparameter tuning of all machine learning models to avoid overfitting and optimize predictive performance. The hyperparameter search ranges for each algorithm were set as follows: RF: number of decision trees (n_estimators) ranging from 50 to 500 with a step of 50, maximum tree depth (max_depth) ranging from 3 to 12, minimum samples for leaf nodes (min_samples_leaf) set to 1, 2, 3; the optimal combination was n_estimators = 300, max_depth = 7, min_samples_leaf = 2. Logistic regression: regularization strength parameter (C) searched over 0.01, 0.1, 1, 10, 100, with L2 penalty adopted; the optimal C value was 1. Support vector machine: kernel function selected among linear, rbf, poly; penalty coefficient (C) from 0.01 to 100, kernel width coefficient (gamma) from 0.001 to 1; the optimal configuration was rbf kernel, *C* = 10, gamma = 0.01. All candidate hyperparameter combinations were evaluated by cross-validated AUC, and the group with the highest mean AUC across 10 folds was selected as the final hyperparameter configuration for each model.

**Figure 2:**
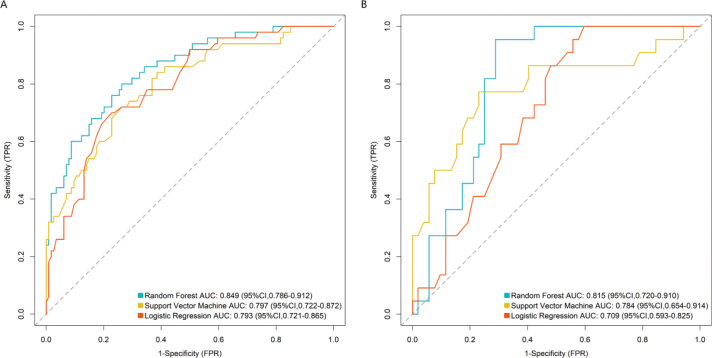
Receiver Operating Characteristic (ROC) curve analysis of the prediction model in the training (A) and validation (B) sets. AUC: area under the receiver operating characteristic curve.

**Figure 3:**
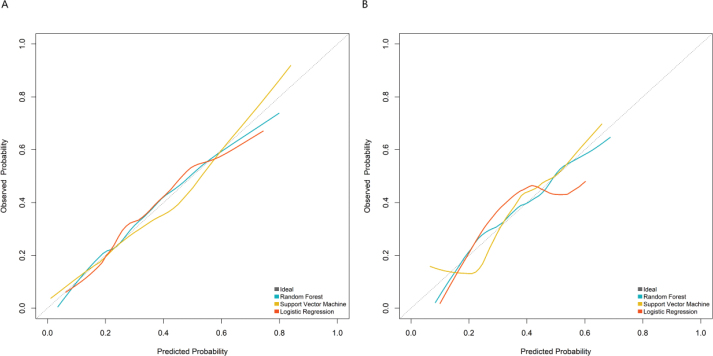
Calibration curve analysis of the prediction model in the training (A) and validation (B) sets.

**Figure 4:**
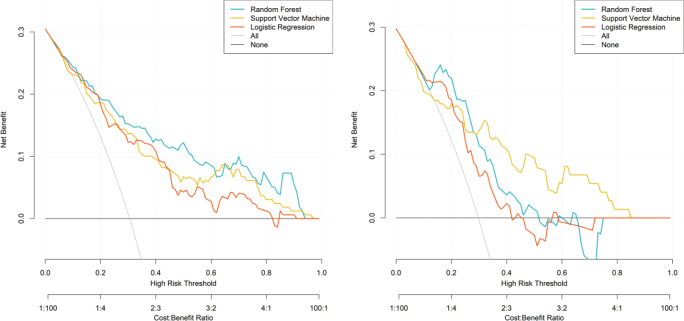
Clinical decision curve analysis of the prediction model in the training (A) and validation (B) sets.

Based on the core predictive variables identified by univariate analysis and LASSO regression, three machine learning prediction models – RF, support vector machine, and logistic regression – were constructed to systematically evaluate the predictive efficacy for adverse gingival embrasure changes after clear aligner treatment. By comparing the discriminative ability of each model in the training and validation sets, the results demonstrated that the RF model achieved the optimal overall predictive performance. In the training cohort, the RF model yielded an AUC of 0.849 (95% CI: 0.786–0.912). In the validation set, its AUC was 0.815 (95% CI: 0.720–0.910). The logistic regression model showed an AUC of 0.793 (95% CI: 0.721–0.865) in the training set and 0.709 (95% CI: 0.593–0.825) in the validation set. The support vector machine model achieved an AUC of 0.797 (95% CI: 0.722–0.872) in the training set and 0.784 (95% CI: 0.654–0.914) in the validation set (Figure 2). DeLong test was used for pairwise comparison of AUC values among the three models. In the training set, the AUC of RF was significantly higher than that of logistic regression (*Z* = 2.132, *p* = 0.033) and support vector machine (*Z* = 1.987, *p* = 0.047), while no significant difference was observed between logistic regression and support vector machine. In the validation set, the AUC of RF remained significantly higher than that of support vector machine (*Z* = 2.156, *p* = 0.031) and logistic regression (*Z* = 2.409, *p* = 0.016). Overall, the RF model presented the best discriminative ability in both the training and validation sets. Consequently, the RF model, which exhibited the highest and most stable AUC in both sets, was identified as the optimal model for predicting gingival embrasure risk after clear aligner treatment. Furthermore, calibration curve analysis (Figure 3) revealed that in the training set, the predicted probability curve of the support vector machine model most closely aligned with the ideal diagonal line. In the validation set, the curves of logistic regression and support vector machine deviated obviously from the diagonal, while the RF model maintained good calibration performance. The Brier score was adopted for quantitative calibration assessment, with a lower value indicating smaller deviation between predicted and observed risk probability and superior calibration. In the training cohort, the Brier scores were 0.116 for RF, 0.104 for support vector machine, and 0.128 for logistic regression, with support vector machine showing the best calibration. In the validation cohort, however, the Brier scores were 0.132 for RF, 0.157 for support vector machine, and 0.165 for logistic regression. Although support vector machine performed best in the training set, its calibration substantially worsened in the validation set (Brier score increased by 0.053). In contrast, RF maintained stable calibration across both datasets, with only a minimal increase of 0.016, demonstrating superior calibration stability on external validation data. Additionally, DCA (Figure 4) confirmed that across a wide range of clinically relevant risk thresholds, applying the RF model for gingival embrasure risk prediction yielded significantly higher net clinical benefit compared to the extreme strategies of ‘intervening for all patients’ or ‘intervening for none’. This net benefit was consistently observed in both the training and validation sets, demonstrating superior clinical utility over the logistic regression and support vector machine models. In summary, the RF prediction model, constructed by integrating multimodal data in this study, demonstrates robust discrimination (training set AUC = 0.849, validation set AUC = 0.815), reliable calibration, and substantial net clinical benefit for predicting adverse gingival embrasure changes after clear aligner treatment. Therefore, it provides an objective and reliable quantitative basis for individualized prediction of gingival embrasure risk post-clear aligner therapy, holding significant potential for clinical translation.

### Evaluation of model prediction interpretability

**Figure 5:**
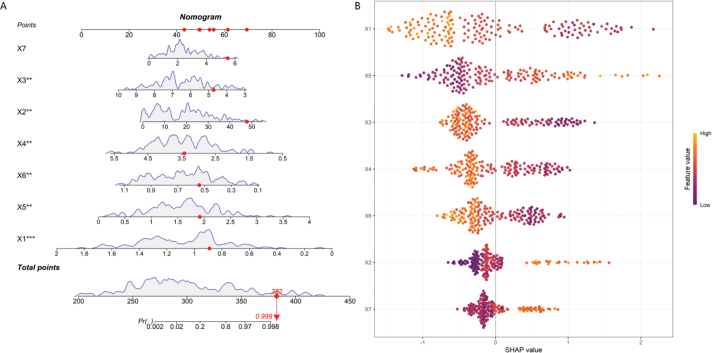
Model interpretability analysis. (A) Nomogram. (B) SHAP feature importance plot. Note: X1: Gingival thickness; X2: Percentage of BOP-positive sites; X3: Proximal contact area; X4: Interdental papilla height; X5: Interproximal alveolar bone height; X6: Buccal/lingual bone plate thickness; X7: Relative movement of adjacent teeth at target site. SHAP: Shapley Additive Explanations; BOP: bleeding on probing.

Based on the seven core predictive variables identified by LASSO regression, a nomogram model for gingival embrasure risk was constructed using the RF algorithm (Figure 5A). This model visually illustrates the contribution and direction of effect of each clinical and radiographic feature on adverse gingival embrasure outcomes following clear aligner treatment. Model results indicated that the percentage of BOP-positive sites (X2), interproximal alveolar bone height (X5), and relative movement of adjacent teeth at the target site (X7) were independent risk factors for predicting high-risk status after clear aligner therapy, with higher values significantly associated with increased risk of adverse post-treatment gingival embrasure changes. Conversely, gingival thickness (X1), proximal contact area (X3), interdental papilla height (X4), and buccal/lingual bone plate thickness (X6) were identified as protective factors, with higher values significantly associated with a low-risk status for gingival embrasure. SHAP analysis further quantified the relative importance of each feature (Figure 5B), ranked in descending order of SHAP value distribution width as follows: gingival thickness (X1), interproximal alveolar bone height (X5), proximal contact area (X3), interdental papilla height (X4), buccal/lingual bone plate thickness (X6), percentage of BOP-positive sites (X2), and relative movement of adjacent teeth at the target site (X7). The mean absolute SHAP value, a quantitative indicator reflecting feature predictive contribution, was calculated for each predictor with corresponding 95% confidence intervals (CI). The 95% confidence intervals for mean absolute SHAP values were estimated using bootstrap resampling with 1000 iterations. Gingival thickness (X1) exhibited the strongest predictive contribution, with a mean absolute SHAP value of 0.872 (95% CI: 0.815–0.929). The interproximal alveolar bone height (X5) ranked second, with a mean absolute SHAP value of 0.641 (95% CI: 0.594–0.688). The mean absolute SHAP values (95% CI) of the remaining variables were listed sequentially: proximal contact area (X3): 0.526 (0.483–0.569), interdental papilla height (X4): 0.418 (0.377–0.459), buccal/lingual bone plate thickness (X6): 0.335 (0.301–0.369), percentage of BOP-positive sites (X2): 0.247 (0.216–0.278), and relative movement of adjacent teeth at the target site (X7): 0.163 (0.138–0.188). The quantitative SHAP results were consistent with the feature importance ranking visualized in the SHAP dependence plot (Figure 5B), confirming that gingival thickness was the dominant risk factor for adverse gingival embrasure changes after invisible orthodontic treatment.

## Discussion

This study focused on adverse changes in gingival embrasures following clear aligner therapy as the primary clinical outcome. Using a retrospective study design, we integrated multimodal data – including periodontal soft tissue characteristics, alveolar bone anatomy, orthodontic force parameters, and oral hygiene status – and successfully developed and validated a predictive model for gingival embrasure risk after clear aligner treatment. The modeling process incorporated univariate analysis, LASSO regression, multivariate logistic regression, and multiple machine learning algorithms. The results showed that the RF model achieved the best predictive performance, with an AUC of 0.849 in the training set and 0.815 in the validation set. This model demonstrated good discriminative ability, calibration, and clinical net benefit. Furthermore, SHAP analysis elucidated the contribution weights and direction of effects of each core variable, providing an interpretable and clinically applicable quantitative tool for individualized risk assessment.

This study identified gingival thickness, buccal/lingual alveolar bone plate thickness, interdental papilla height, and proximal contact area as independent protective factors against gingival embrasure risk. These findings are highly consistent with those reported in previous literature. The gingiva and alveolar bone plate constitute the biological support basis of the interdental papilla. Patients with thin gingival biotype and thin bone plate have poor periodontal tissue resilience and limited blood supply, making them more prone to gingival recession and gap formation during tooth movement [11, 12]. Interdental papilla height and proximal contact area directly determine the initial filling state of the interdental space. A larger contact area and greater papilla height are associated with a lower risk of black triangles [13, 14]. These results suggest that clinical assessment should prioritize the thickness of periodontal soft and hard tissues and papilla morphology in the anterior region. Preventive strategies should be implemented early in patients with thin gingiva, thin bone plate, or short papillae.

Meanwhile, the percentage of BOP-positive sites, interproximal alveolar bone height, and relative movement of adjacent teeth at the target site were identified as independent risk factors. BOP reflects the burden of periodontal inflammation, which can disrupt periodontal fiber attachment and hinder tissue remodeling, thereby significantly increasing the risk of papillary recession after orthodontic treatment [15]. Reduced interproximal alveolar bone height (i.e. increased distance from the cementoenamel junction) indicates insufficient bony support and serves as a key anatomical basis for gingival embrasure formation [16]. Excessive relative movement of adjacent teeth at the target site causes tooth movement to outpace periodontal tissue remodeling, leading to mechanical separation and papillary collapse. This finding aligns with the biological principle of light and slow force application in clear aligner therapy [17]. By incorporating orthodontic force parameters into the predictive model, this study overcomes the limitations of traditional approaches that focus only on anatomical factors, thereby better reflecting real-world clinical decision-making scenarios.

In terms of model performance, this study compared logistic regression, support vector machines, and RF models and confirmed that the RF model is more suitable for handling multimodal, high-dimensional dental data. Conventional logistic regression has limited capacity to fit nonlinear relationships. In contrast, the RF model captures interactions among variables and is less prone to overfitting, maintaining a stable AUC in the validation set. Consequently, this model is more appropriate for clinical application [18, 19]. Pairwise comparison via DeLong test further verified that the AUC of the RF model was significantly higher than that of the logistic regression and support vector machine in both training and validation sets (*p* < 0.05). Calibration curves and DCA further demonstrated that the predicted probabilities of this model closely approximate actual risk and that the model provides clinical net benefit across a wide range of threshold probabilities. Thus, the model can effectively support stratified management strategies – early intervention for event-group patients and routine monitoring for non-event-group patients [20].

The SHAP interpretability analysis addressed the ‘black box’ issue commonly associated with machine learning models. It clarified that gingival thickness and interproximal alveolar bone height are the most influential features. This consistency not only enhances model credibility but also provides clear clinical intervention targets: preoperatively, augmenting gingival thickness, controlling inflammation, and preserving bony support; intraoperatively, controlling adjacent tooth displacement and optimizing movement paths; and postoperatively, reinforcing periodontal maintenance and papilla monitoring [21]. This complete ‘prediction–interpretation–intervention’ loop elevates the model from a statistical tool to a clinical decision support system.

This study has several limitations. First, it was a single-center retrospective study, and selection bias and information bias cannot be completely ruled out. In this study, cases with more than 20% missing data of core indicators were excluded, and a small amount of missing demographic data was supplemented by mean imputation, which may further introduce selection bias. Future multicenter prospective cohort studies are needed for external validation and to reduce relevant bias. Second, behavioral variables such as patient oral hygiene compliance, aligner wear time, and follow-up visit frequency were not included; incorporating these variables could further improve model accuracy. Third, the sample predominantly consisted of adult patients, whereas adolescents have stronger periodontal remodeling capacity; thus, the generalizability of the model may be limited. Fourth, this study focused on gingival embrasures in the anterior region; risk characteristics and predictive factors for posterior embrasures warrant separate investigation. Fifth, we adopted the planned adjacent tooth movement rather than the actual movement for analysis. Since the actual tooth movement is usually lower than the planned value in clinical treatment, relevant estimation bias may exist. Sixth, the model only completed internal validation and lacked independent external dataset validation, which limited its generalization ability. In addition, although the EPV met the basic standard, the limited number of positive events in the training set may still bring potential risks of model instability.

In conclusion, the multimodal RF risk prediction model developed in this study can accurately identify individuals at high risk for gingival embrasure changes before clear aligner therapy. It quantifies the risk contribution of each factor and provides an objective basis for treatment planning, early intervention, and patient–clinician communication. By integrating multidimensional indicators – anatomical, mechanical, and inflammatory – this model achieves both accuracy and interpretability, can assist clinicians in quantitative risk assessment, and aligns with the trend toward precision medicine in orthodontics. Further external validation and comparison with clinical manual assessment are required before large-scale clinical application.

## Supplementary Material



## Data Availability

All data that support the findings of this study are available from the corresponding authors upon reasonable request.
