# Supplementary material for: Predicting gingival embrasure risk after invisible orthodontics using multimodal data and machine learning
Source: Acta Odontol Scand. 2026 Jul 22;85:46562. doi: 10.2340/aos.v85.46562 (PMC13401248; doi:10.2340/aos.v85.46562)
Supplement: Supplementary file 1 [file AOS-85-46562-s1.pdf]

Supplementary material has been published as submitted. It has not been copyedited or typeset by Acta Odontologica Scandinavica.

Supplemental Table 1. Variable assignment

Variable	Meaning	Assignment
X1	Gingival thickness	Continuous variable
X2	Percentage of BOP-positive sites	Continuous variable
X3	Proximal contact area	Continuous variable
X4	Interdental papilla height	Continuous variable
X5	Interproximal alveolar bone height	Continuous variable
X6	Buccal/lingual bone plate thickness	Continuous variable
X7	Relative movement of adjacent teeth at target site	Continuous variable
Y	Gingival embrasure risk after clear aligner treatment	event group=1 ， non-event group=0
